# Circulating CD137^+^ T Cell Levels Are Correlated with Response to Pembrolizumab Treatment in Advanced Head and Neck Cancer Patients

**DOI:** 10.3390/ijms24087114

**Published:** 2023-04-12

**Authors:** Alessio Cirillo, Ilaria Grazia Zizzari, Andrea Botticelli, Lidia Strigari, Hassan Rahimi, Simone Scagnoli, Fabio Scirocchi, Angelina Pernazza, Angelica Pace, Bruna Cerbelli, Giulia d’Amati, Paolo Marchetti, Marianna Nuti, Aurelia Rughetti, Chiara Napoletano

**Affiliations:** 1 Division of Oncology, Department of Radiological, Oncological and Pathological Science, Policlinico Umberto I, “Sapienza” University of Rome, 00161 Rome, Italy; 2 Laboratory of Tumor Immunology and Cell Therapies, Department of Experimental Medicine, “Sapienza” University of Rome, 00161 Rome, Italychiara.napoletano@uniroma1.it (C.N.); 3 Medical Physics Unit, “Sant’Orsola-Malpighi” Hospital, 40138 Bologna, Italy; 4 Department of Radiology, Oncology and Pathology, “Sapienza” University of Rome, 00161 Rome, Italy; 5 Istituto Dermopatico dell’Immacolata (IDI-IRCCS), 00161 Rome, Italy

**Keywords:** immunotherapy, anti-PD1, HNSCC, CD137, 4-1BB

## Abstract

Pembrolizumab, an anti-PD-1 antibody, has been approved as first-line treatment for recurrent or metastatic head and neck squamous cell carcinoma ((R/M) HNSCC). However, only a minority of patients benefit from immunotherapy, which highlights the need to identify novel biomarkers to optimize treatment strategies. CD137^+^ T cells have been identified as tumour-specific T cells correlated with immunotherapy responses in several solid tumours. In this study, we investigated the role of circulating CD137^+^ T cells in (R/M) HNSCC patients undergoing pembrolizumab treatment. PBMCs obtained from 40 (R/M) HNSCC patients with a PD-L1 combined positive score (CPS) ≥1 were analysed at baseline via cytofluorimetry for the expression of CD137, and it was found that the percentage of CD3^+^CD137^+^ cells is correlated with the clinical benefit rate (CBR), PFS, and OS. The results show that levels of circulating CD137^+^ T cells are significantly higher in responder patients than in non-responders (*p* = 0.03). Moreover, patients with CD3^+^CD137^+^ percentage ≥1.65% had prolonged OS (*p* = 0.02) and PFS (*p* = 0.02). Multivariate analysis, on a combination of biological and clinical parameters, showed that high levels of CD3^+^CD137^+^ cells (≥1.65%) and performance status (PS) = 0 are independent prognostic factors of PFS (CD137^+^ T cells, *p* = 0.007; PS, *p* = 0.002) and OS (CD137^+^ T cells, *p* = 0.006; PS, *p* = 0.001). Our results suggest that levels of circulating CD137^+^ T cells could serve as biomarkers for predicting the response of (R/M) HNSCC patients to pembrolizumab treatment, thus contributing to the success of anti-cancer treatment.

## 1. Introduction

Head and neck squamous cell carcinoma (HNSCC) is a heterogeneous disease of the upper aerodigestive tract (oral cavity, nasopharynx, oropharynx, hypopharynx, and larynx) including different anatomical sites, histological sites, and HPV^+^ and HPV^−^ cancers. HNSCC patients treated with combinatory therapies (surgery, chemotherapy, and radiation) generally relapse within 3 to 5 years [1,2]. In recent years, the introduction of immunotherapy has shown survival benefits, demonstrating the efficacy of targeting immune cells [3,4,5,6]. Of the various anti-PD1 treatments, nivolumab has been approved by the FDA for treating recurrent/metastatic (R/M) HNSCC in patients whose disease has progressed during or after platinum-based chemotherapy [3]. Most recently, pembrolizumab, an anti-PD1 antibody, was approved as an upfront treatment for HNSCC, completely changing the management of the affected patients. The phase III clinical trial Keynote 048 sanctioned the introduction of this agent as first-line treatment for patients with (R/M) HNSCC with a PD-L1 combined positive score (CPS) ≥1 [7]. Pembrolizumab, alone or in combination with platinum-based chemotherapy, significantly improves the overall survival (OS) of (R/M) HNSCC patients with CPS ≥1 or ≥20 compared with chemotherapy plus cetuximab, an anti-EGFR monoclonal antibody. Despite the encouraging results, only a limited number of patients experienced long-term benefits, highlighting the urgent need to improve patient selection.

The evaluation of PD-L1 on both tumour and immune cells defined by CPS represents the only parameter currently used by clinicians to guide therapeutic choices. However, PD-L1 is a dynamic biomarker, and its role as a predictive factor remains controversial. Several lines of evidence show conflicting results regarding the prognostic significance of PD-L1 in head and neck cancer in the context of cellular expression, tissue localization, and protein forms [8,9]. Moreover, PD-L1-positive patients who receive anti-PD1/PD-L1 immunotherapy seem to have an increased overall response rate (ORR) and favourable OS at 6 and 12 months from the start of treatment [10]. Other authors have demonstrated that high PD-L1 expression in head and neck cancer patients is not related to OS, but correlated with several clinicopathological features, such as sex, histological differentiation, distant metastasis, and HPV status, thus appearing to be indicative of better patient prognosis [11,12]. Although the PD-1/PD-L1 axis plays an important role in the initial phase of tumour escape and progression [13], several other immune parameters influence patient survival, and have been considered as possible predictors in HNSCC alone or in combination with PD-L1 expression [14]. The immune infiltrate of the tumour microenvironment (TME) is the most exploited; its composition seems to have a fundamental role in dictating patient survival. Indeed, the presence of tumour-infiltrating lymphocytes (TILs) is associated with better prognosis, suggesting that the number of TILs has an important impact on the control of tumour growth [15,16]. On the contrary, the presence of high levels of immunosuppressive cells, such as fibroblast-associated fibroblasts (CAFs) [17] or myeloid-derived suppressive cells (MDSCs) is correlated with worse prognosis [18], whereas a high level of regulatory T cells seems to prolong patient survival [19]. However, data regarding the analysis of circulating immune cells in HNSCC are scarce. We recently demonstrated the importance of the circulating CD137^+^ T cell subset as a predictive biomarker of response to immunotherapy [20]. CD137 (4-1BB) is a molecule belonging to the TNFR-family, expressed by activated T cells and by several types of immune and non-immune cells [21,22]. The binding between CD137 and its ligand (CD137L), expressed by antigen-presenting cells (APCs), induces bidirectional signalling. In lymphocytes, this binding also enhances the effector functions of CD8^+^ T cells by promoting cytotoxic activity and cytokine release (IFNγ, TNFα, and IL2), and of CD4^+^ lymphocytes by inducing the production of Th1 cytokines. In APC, it enhances the capacity for antigen presentation [23,24].

This marker also identifies the naturally occurring tumour-specific T cells in mice and humans [25], highlighting the importance of the CD137^+^ T cell subset in the induction of an effective anti-tumour immune response. Indeed, we and others have shown that high levels of infiltrating and circulating CD137^+^ T cells correlate with improved survival in lung, ovarian, and liver cancer patients [25,26,27] and with the response to anti-PD-1 treatment [20].

In this study, we analysed the levels of circulating CD137^+^ T cells in (R/M) HNSCC patients who underwent pembrolizumab treatment as a first-line therapy, alone or in combination with platinum-based chemotherapy. The frequency of CD137^+^ T cells evaluated before immunotherapy began was found to be correlated with the clinical parameters of the clinical benefit rate (CBR), progression-free survival (PFS), and OS. Our results indicate that circulating CD137^+^ T cells could represent an optimal biomarker to define the response of (R/M) HNSCC patients to pembrolizumab treatment.

## 2. Results

### 2.1. Patient Characteristics

Forty patients with recurrent or metastatic HNSCC were enrolled in this study (Table 1). All patients had CPS ≥ 1 and underwent pembrolizumab as monotherapy (67%) or pembrolizumab plus chemotherapy (33%). Before starting anti-PD1 treatment, 38% of patients were scored as performance status (PS) = 0 and 42% as PS = 1. Only eight patients (20%) were defined as PS = 2. The oral cavity was the primary tumour site in most patients (60%), followed by the larynx (20%), oropharynx (10%), salivary glands (8%), and nasopharynx (2%). CPS ≥ 20 was observed in 58% of tumour samples. Most patients were HPV-negative (95%) and current or former smokers (75%); approximately half of the patients (58%) denied alcohol consumption. The clinical benefit rate (CBR) was used to classify responder (R) (38%) and not-responder (NR) (62%) patients to an anti-PD-1 treatment. A total of 25 patients suffered disease progression 6 months after beginning immunotherapy, while 5 and 10 patients showed a stable or partial response, respectively. The median PFS and OS were 3.5 and 10 months, respectively.

### 2.2. High Circulating Levels of CD137^+^ T Cells Are Associated with Response to Pembrolizumab Treatment

**Figure 1:**
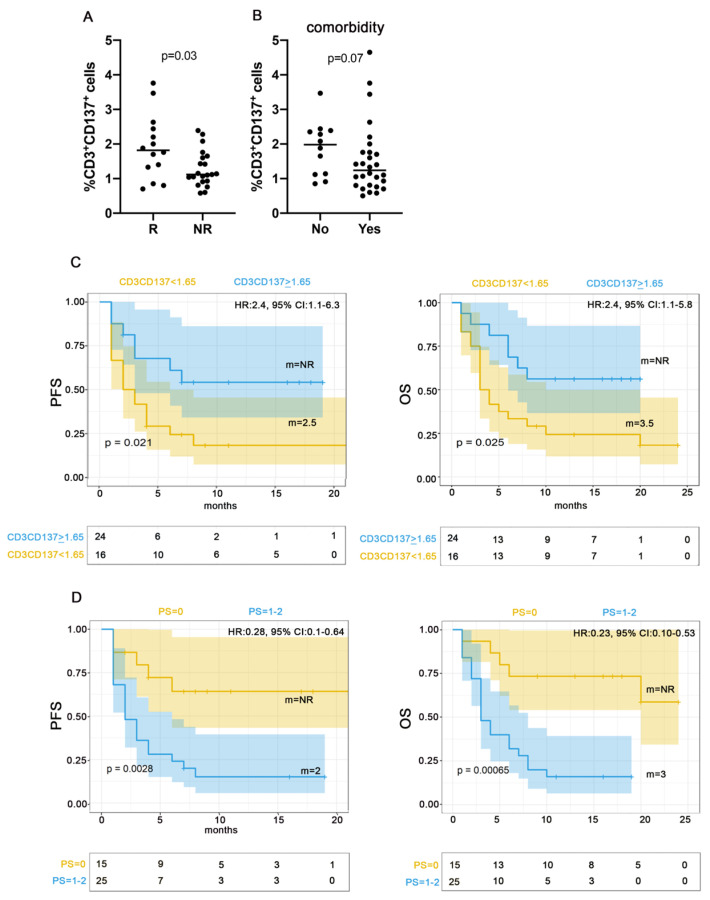
CD137^+^ T cells are correlated with response to anti–PD–1 treatment and patient survival. (**A**) The scattered dot plot shows the values of CD3^+^CD137^+^ cells in responder (R) and non–responder (NR) patients ± standard deviation (SD). The horizontal lines correspond to the median values of CD3^+^CD137^+^ lymphocytes of the two groups. (**B**) The scattered dot plot represents the values of CD3^+^CD137^+^ cells in patients with (Yes) or without (No) comorbidities. The horizontal lines correspond to the median values of CD3^+^CD137^+^ lymphocytes of the two groups. (**C**) Kaplan–Meier curves for PFS and OS were used to determine 1.65% as the cut-off of circulating CD3^+^CD137^+^ cells wherein patients with a percentage ≥1.65% showed prolonged survival. (**D**) Kaplan–Meier curves for PFS and OS considering the score related to performance status (PS) (PS = 0 vs. PS1 = 1, 2). Patients with PS = 0 showed a longer PFS and OS than those with PS = 1, 2. m = Months; NR = not yet reached.

To understand the role of circulating CD137^+^ T cells in (R/M) HNSCC patients undergoing pembrolizumab treatment, we evaluated the levels of CD137^+^ T cells derived from the peripheral blood of the 40 cancer patients before beginning immunotherapy. The results demonstrate that high levels of circulating CD137^+^ T cells are correlated with (R/M) HNSCC patients’ response to pembrolizumab treatment (*p* = 0.03) (Figure 1A; gating strategies in Figure S1A). Indeed, responding patients showed a significantly higher percentage of CD3^+^CD137^+^ than non-responders (R vs. NR; 1.9 ± 0.24 vs. 1.2 ± 0.1, *p* = 0.03). This difference could not be ascribed to CD8^+^CD137^+^ or CD4^+^CD137^+^ T cells, which did not show any significant association with clinical response (CD8^+^CD137^+^: R vs. NR: 0.9 ± 0.1 vs. 0.78 ± 0.1, *p* = 0.5; CD4^+^CD137^+^: R vs. NR: 0.9 ± 0.1 vs. 0.73 ± 0.1, *p* = 0.3) (Figure S1B). Moreover, no difference in the percentage of CD137^+^ T cells was found when patients were analysed according to treatment (pembrolizumab alone vs. pembrolizumab plus chemotherapy, *p* = 0.3).

The levels of CD137^+^ T cells were also found to be correlated with several clinical parameters, i.e., PS, presence of visceral metastasis, CPS ≥ 1, CPS ≥ 20, comorbidities, toxicity, and previous therapies. No significant correlation was found between the number of CD137^+^ T cells and any of these parameters; however, the patients with no comorbidity tended to have higher levels of CD137^+^ T than those with worse clinical status (Figure 1B) (no comorbidity vs. comorbidity: 1.9 ± 0.2 vs. 1.2 ± 0.2, *p* = 0.07). All these data suggest that the subset of CD137^+^ T cells could be considered as a potential biomarker for the response of (R/M) HNSCC patients to pembrolizumab treatment and serve as a useful tool to identify patients for whom pembrolizumab treatment would improve clinical status.

### 2.3. CD137^+^ T Cells as a Predictive and Prognostic Factor of PFS and OS in (R/M) HNSCC Patients

The levels of circulating CD137^+^ T cells and several clinical parameters, such as age, sex, CPS value, PS, and presence of visceral metastasis, were further examined by univariate analysis to predict survival (Table 2 and Table 3). Concerning the analysis of CD3^+^CD137^+^ cells, a cut-off of 1.65% was found. The identified cut-off values for OS and PFS correspond to the minimum *p*-values (OS, *p* = 0.02; PFS, *p* = 0.02). The numbers of patients having CD137 values ≥ or <1.65 were 24 (60%) and 16 (40%), respectively. Patients with a percentage of CD137^+^ T cells ≥ 1.65% showed an increase in PFS (CD137 ≥ 1.65% vs. CD137 < 1.65: median survival was not reached vs. 2.5 months, *p* = 0.02) and OS (CD137 ≥ 1.65% vs. CD137 < 1.65: median survival was not reached vs. 3.5 months, *p* = 0.02) (Figure 1C). Moreover, univariate analysis of clinical parameters showed that PS was the only parameter associated with prolonged PFS and OS. Patients scored as PS = 0 had a longer PFS (PS = 0 vs. PS = 1, 2: median survival was not reached vs. 2 months, *p* = 0.003) and OS (PS = 0 vs. PS = 1, 2: median survival was not reached vs. 3 months, *p* = 0.0006) compared to patients with PS = 1, 2 (Figure 1D).

Multivariate analysis revealed that PS = 0 and the percentage of CD137^+^ T cells ≥ 1.65% are two independent prognostic factors of PFS and OS (Table 2 and Table 3).

## 3. Discussion

The immune landscape of (R/M) HNSCC patients is characterised by strong immunosuppression and several dysfunctional immune cells [28]. Its immune complexity is further conferred by the TME heterogeneity detected for distinct tumour locations and HPV infection [28,29]. These differences are reflected in different susceptibilities to therapy and patient survival [30], although in this setting patients usually have poor prognosis with a low survival rate. The introduction of immunotherapy as upfront treatment for (R/M) HNSCC has shown survival benefits, demonstrating the importance of better understanding the state of these patients’ immune systems.

In this study, we propose that the levels of CD137^+^ T cells prior to immunotherapy, as a biomarker of immune activation, is able to predict the response and clinical outcome of (R/M) HNSCC patients to pembrolizumab treatment. Moreover, we identify the CD137^+^ T cell subset as an independent prognostic factor of survival, suggesting that the presence of this immune population represents a crucial point for successful anti-PD-1 immunotherapy administered as first-line treatment. Interestingly, by combining the levels of CD137^+^ T cells and PS, it is possible to better define the profile of (R/M) HNSCC patients with longer survival.

In line with our results, several other studies have identified circulating CD137^+^ T cells as predictive biomarkers in different cancer settings. In advanced renal carcinoma, CD137^+^ lymphocytes are a predictor of the clinical response to tyrosine kinase inhibitors [31]. In non-small cell lung carcinoma, patients with early progression show decreased levels of circulating CD137^+^ T cells, together with high levels of the IgM-rheumatoid factor [26]. In melanoma patients, CD137^+^CD8^+^ T cells are associated with a disease-free status [32]. In addition, we recently demonstrated and validated, in a cohort of 109 patients with different metastatic solid tumours, that high levels of circulating CD137^+^ T cells (cut-off: 1.2%) are a prognostic factor for PFS and OS, and that CD8^+^CD137^+^ T cells are a prognostic factor for PFS. We also showed that patients with a complete response to anti-PD-1 immunotherapy had high levels of CD137^+^ cells in the tumour microenvironment within tertiary lymphoid structures surrounding the tumour mass [20]. In the present study, the critical cut-off value of CD137^+^ T cells for indicating survival was identified as 1.65%. Moreover, the analysis carried out on CD8^+^CD137^+^ and CD4^+^CD137^+^ T cells revealed that these cellular subsets, examined as single populations, do not seem to have a particular impact on the induction of an anti-tumour immune response. These data disagree with those of several authors’ suggesting a primarily role for CD137 in CD8 T cells [25,27]. However, several others demonstrated that both CD8^+^CD137^+^ and CD4^+^CD137^+^ T cells contribute, with similar efficacy, in responses against tumours. Indeed, triggering the CD137 pathway promotes the development of cytotoxic activity in CD8^+^ T cells and the induction of a Th1 response in CD4 T cells, which boosts effector anti-tumour functions [24,33,34]. All this evidence suggests that CD137^+^ T cell levels and the prevalence of a specific CD137^+^ T cell subset can vary according to both the tumour’s histotype and the TME. In particular, in HNSCC, high levels of activated tumour-specific T cells are required to overcome the immune suppression induced by tumour mass, and the synergistic involvement of both CD8^+^CD137^+^ and CD4^+^CD137^+^ T cells is particularly important in this type of tumour, which is characteristic of a highly immunosuppressive milieu [29].

Moreover, most patients (95%) analysed in this study were HPV-negative. These patients had worse prognoses, and were characterised by a TME with a low number of TILs and high infiltration of immunosuppressive cells, confirming the need to strongly activate the immune response to obtain an efficacious immune response. In addition, the cut-off value of 1.65% for CD137^+^ T cells was used to analyse the survival rates of patients treated with pembrolizumab and pembrolizumab plus chemotherapy CD137^+^ T cells seemed to distinguish patients with a longer OS only in the pembrolizumab group. However, the difference in the pembrolizumab plus chemotherapy patients was not found to be statistically significant due to the limited number of patients included in this group.

The fundamental role of the CD137^+^ T cell subset in the induction of anti-tumour immunity was further demonstrated in earlier studies of mice models in which agonistic anti-CD137 antibodies were employed. In those studies, antibodies were demonstrated to increase the levels of anti-tumour-specific memory T cells, induce a long-lasting immune response [35], and reduce immunosuppression by decreasing the amount of regulatory Tregs and MDSCs [36,37]. In addition, clinical studies have demonstrated that the agonistic monoclonal antibody anti-CD137, urelumab, triggers the activation of IFNγ signalling and pro-inflammatory cytokines [38]. All this evidence highlights the importance of CD137^+^ T cells as key contributors to the anti-tumour immune response and as a novel protagonist of immune-based approaches.

To date, PD-L1 CPS is the most widely used biomarker for guiding the selection of (R/M) HNSCC patients for treatment based on predicted response, although with contradictory evidence. Its role as a biomarker has been demonstrated in different phase III clinical trials (KEYNOTE 040 and KEYNOTE 048) [7,39], in which it was observed that PD-L1^+^ patients showed increased survival. However, several other studies have shown similar therapeutic benefits for both PD-L1^+^ and PD-L1^−^ patients, with no significant difference in overall survival, suggesting that PD-L1 alone does not adequately distinguish which patients will benefit from treatment [40,41]. In our study, all patients were PD-L1 CPS-positive and underwent immunotherapy. However, most patients (62%) were non-responders with poor survival, confirming that in our cohort of patients, the expression of PD-L1 alone was not sufficient for optimal patient selection.

Discordant results across studies could be ascribed to several factors. The most relevant is linked to the absence of uniformity in the assays and variability in the threshold [42,43]. Different PD-L1 antibodies have been used in the numerous assays with significant variation in the percentage of positive immune and tumour cells [44]. Moreover, the current guidelines do not specify the timing of the analysis, the location of biopsy, or the volume of tumour samples associated with the incorrect classification of PD-L1 expression in tumour tissue. In addition, PD-L1 signalling can be regulated by several pathways, such as PI3K, Akt/PKB, and MAPK; these are frequently altered in HNSCC patients, leading to PD-L1 being subjected to extreme temporal variation and spatial heterogeneity [45,46,47]. This heterogeneity could also be dependent on any previous therapy, such as chemotherapy, that increases PD-L1 levels [48].

Beyond PD-L1 CPS, most potential biomarkers examined in HNSCC are derived from tumour tissue analysis [14]. The major limitation of these approaches is the availability of material that cannot be analysed over time. Instead, the analysis of circulating CD137^+^ T cells overcomes all of these critical issues and provides information regarding the activation state of a patient’s immune system in real time. Moreover, consecutive blood withdrawals could be used to predict the efficacy of therapy and to monitor disease development. This analysis can be also standardised and easily employed in a hospital setting to monitor the immune fitness of cancer patients during anti-tumour therapy.

In conclusion, in this study, we identified CD137^+^ T cells as a potential biomarker to predict the success of pembrolizumab treatment and longer survival in (R/M) HNSCC patients. Although the number of patients was limited, these results are particularly important in this patient group, in which PD-L1 CPS does not represent a reliable immunological parameter for patient selection. We believe that the predominance of this cellular subset could be used by oncologists to monitor the response to pembrolizumab treatment as upfront therapy. Further studies with large cohorts that include nivolumab-treated patients will be conducted to validate these data.

## 4. Materials and Methods

### 4.1. Patients

In this study, the enrolled patients had a confirmed diagnosis of recurrent or metastatic HNSCC and had undergone treatment with single-agent anti-PD-1 or anti-PD-1 associated with chemotherapy as first-line treatment at the Medical Oncology Department of Policlinico Umberto I Hospital. The follow-up was monitored for 24 months. Patients were mainly treated with pembrolizumab or pembrolizumab plus chemotherapy using standard doses and schedules until disease progression or unacceptable toxicity. Toxicity was reported according to Common Terminology Criteria for Adverse Events (version 4.0) and was evaluated on day 1 of every cycle until the end of treatment.

Criteria for inclusion were age >18 years; histologically documented diagnosis of HNSCC of the oral cavity, oropharynx, larynx, salivary glands, and nasopharynx; and EOCG performance status (PS) scored between 0 and 2. Exclusion criteria were autoimmune disease, systemic immunosuppression, and any significant comorbidity. PFS, OS, and CBR were evaluated. PFS was defined as the time immunotherapy began until the first documented tumour progression or death from any cause. OS was defined as the interval between the beginning of immunotherapy to death from any cause. The response was assessed every month until disease progression using immune-related Response Evaluation Criteria in Solid Tumours (i-RECIST) and classified as a complete or partial response and stable or progressive disease. The CBR was used to classify patients as responders (patients with a complete or partial response and stable disease) and non-responders (progressors) after 6 months of therapy. CPS was defined as the number of PD-L1-positive cells, including tumour cells, lymphocytes, and macrophages, divided by the total number of tumour cells × 100. The study was conducted following the Declaration of Helsinki and good clinical practice guidelines. All patients provided signed informed consent (RIF.CE: 4181).

### 4.2. PBMCs Isolation

Peripheral blood mononuclear cells (PBMCs) were isolated from the blood samples of 40 HNSCC patients using Ficoll Hypaque (lympholyte-H, Cedarlane, Burlington, VT, Canada) before the immunotherapy began (T0). Cells were cryopreserved until use.

### 4.3. Cytofluorimetry

PBMC phenotyping was carried out via cytofluorimetry combining the following conjugated anti-human monoclonal antibodies (MoAbs): anti-CD3 BV510 (clone HIT3a), anti-CD8 APC-H7 (clone SK1), and anti-CD137 APC (clone 4B4-1). All antibodies were purchased from Becton Dickinson (San Diego, CA, USA). Live and dead cells were stained using a LIVE/DEAD fixable yellow dead cell staining kit (Invitrogen, Waltham, MA, USA). The autofluorescence of the cells and fluorescence minus one (FMO) were used as negative controls for the expression of CD137. Samples were analysed using a FACSCanto II flow cytometer and analysed by FlowJo (version 10.8.8, Becton Dickinson) analysis software.

### 4.4. Statistical Analysis

Descriptive statistics (median, range, and percentages) of the clinical and biological characteristics of HNSCC patients were analysed. Student’s *t*-test was used to compare two groups of data. The impacts of clinicopathological variables on OS and PFS were analysed via univariate followed by multivariate analyses (UVA and MVA, respectively). With regards to UVA, HNSCC patients’ OS and PFS were analysed using the Kaplan–Meier method and log-rank tests. The optimal cut-off values of CD137 for OS or PFS were those that corresponded to the minimum *p*-value for each endpoint among those calculated using Kaplan–Meier curves, varying the threshold of CD137 from the minimum to the maximum values determined in our cohort. The clinicopathological variables deemed of potential relevance in the univariate analysis (corresponding to a cut-off of *p* < 0.10) were included in the multivariate Cox proportional hazards regression analysis to identify the prognostic variables. The sample size calculation was performed assuming an α level of 0.05 and a β level of 0.20 (power 80%). With these assumptions, the required sample size was 17 cases in each group (i.e., a total of 34 cases) to detect a percentage difference of at least 1.5% between groups, and having standard deviations of 1% and 2%, in the groups with good and poor prognosis, respectively. The simple size was increased to 40 patients to take patient loss at follow-up into consideration.

## Figures and Tables

**Table 1:** Patients’ characteristics.

	n° (%) 40 (100)
**Sex:**	
Male	31 (78)
Female	9 (22)
**Age:**	
Median range	71 (42–87)
< 65	17 (43)
>65	23 (57)
**EOCG Performance Status:**	
0	15 (38)
1	17 (42)
2	8 (20)
**Primary tumour site:**	
Oral cavity	24 (60)
Larynx	8 (20)
Oropharynx	4 (10)
Salivary glands	3 (8)
Nasopharynx	1 (2)
**CPS:**	
>1	40 (100)
>20	23 (58)
**Therapy for metastatic disease:**	
Pembrolizumab	27 (67)
CT+ Pembrolizumab	13 (33)
**HPV status:**	
negative	38 (95)
positive	2 (5)
**Smoking status:**	
smoker (current/former)	30 (75)
non-smoker	10 (25)
**Alcohol consumption:**	
yes (moderate/high)	17 (42)
no	23 (58)
**Response to treatment:**	
yes	15 (38)
no	25 (62)

CPS: PD-L1 combined positive score; CT: chemotherapy.

**Table 2:** Predictive and prognostic factors for progression free survival.

Variables	Univariate Analysis		Multivariate Analysis	
	HR (95%CI)	*p* Value	HR (95%CI)	*p* Value
Age (<71 vs. ≥71)	0.65 (0.24 to 1.44)	0.25		
Sex (Male vs. Female)	0.53 (0.13 to 1.19)	0.1		
CPS ≥ 1	3.18 (0.89 to 222.3)	0.06		
CPS ≥ 20	0.99 (0.4 to 2.43)	0.99		
Metastasis (Yes vs. No)	1.18 (0.5 to 3.03)	0.65		
PS (0 vs. 1, 2)	0.28 (0.1 to 0.64)	**0.003**	2.2 (1.3 to 3.8)	**0.002**
CD3^+^CD137^+^ ≥ 1.65	2.46 (1.1 to 6.3)	**0.02**	0.28 (0.1 to 0.7)	**0.007**

CPS: PDL1 combined positive score; PS: performance status.

**Table 3:** Predictive and prognostic factors for overall survival.

Variables	Univariate Analysis		Multivariate Analysis	
	HR (95%CI)	*p* Value	HR (95%CI)	*p* Value
Age (<71 vs. ≥71)	0.58 (0.24 to 1.29)	0.17		
Sex (Male vs. Female)	0.488 (0.14 to 1.13)	0.08		
CPS ≥ 1	3.66 (0.94 to 186.8)	0.05		
CPS ≥ 20	1.01 (0.44 to 2.31)	0.9		
Metastasis (Yes vs. No)	1.16 (0.51 to 2.7)	0.7		
PS (0 vs. 1, 2)	0.23 (0.1 to 0.53)	**0.0006**	2.29 (1.39 to 3.78)	**0.001**
CD3^+^CD137^+^ ≥1.65	2.24 (1.12 to 5.8)	**0.02**	0.28 (0.11 to 0.69)	**0.006**

CPS: PDL1 combined positive score; PS: performance status.

## Data Availability

Not applicable.

## Supplementary Materials

The following supporting information can be downloaded at https://www.mdpi.com/article/10.3390/ijms24087114/s1.
