# Supplementary material for: Circulating CD137+ T Cell Levels Are Correlated with Response to Pembrolizumab Treatment in Advanced Head and Neck Cancer Patients
Source: Int J Mol Sci. 2023 Apr 12;24(8):7114. doi: 10.3390/ijms24087114 (PMC10138766; doi:10.3390/ijms24087114)
Supplement: Supplementary file 1 [file ijms-24-07114-s001.zip › ijms-2289128-supplementary.pdf]

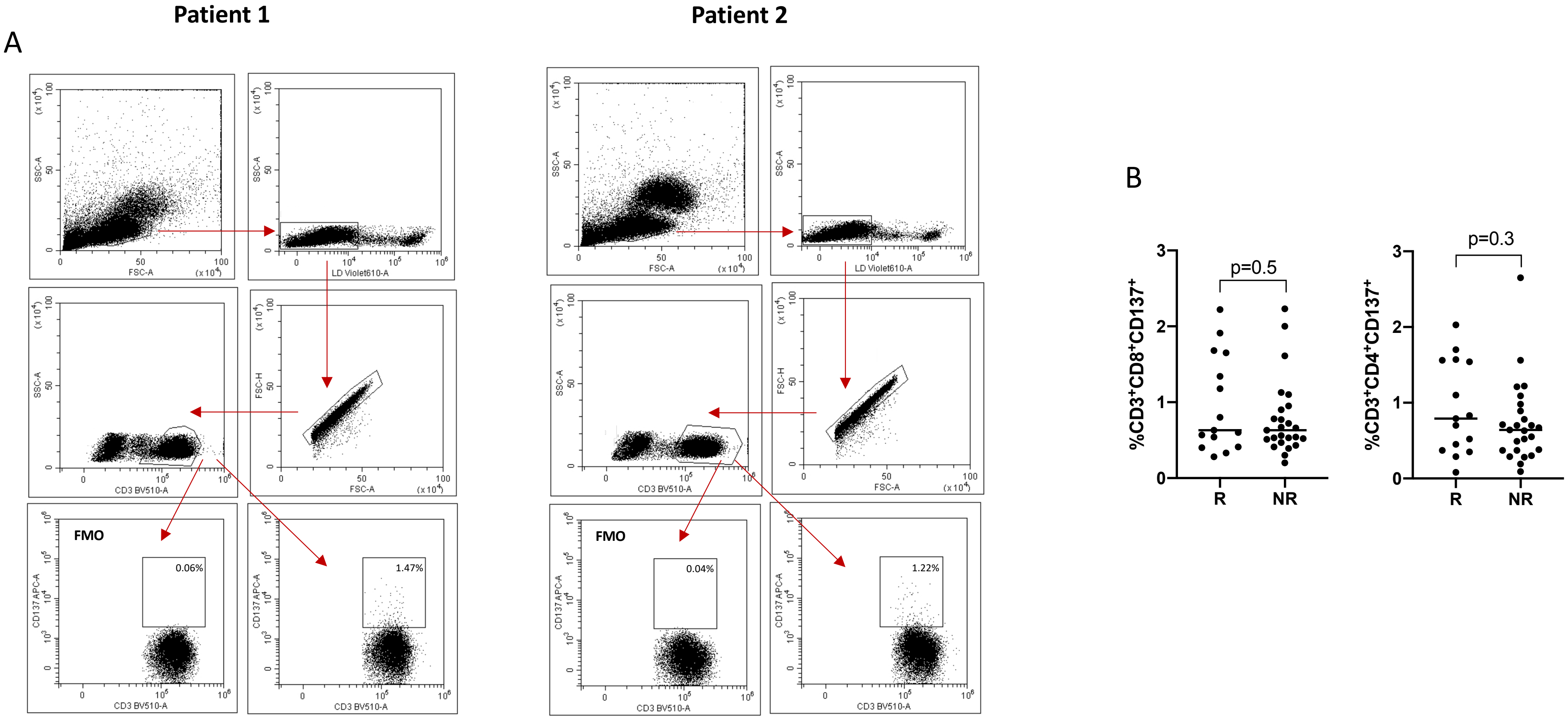

Figure S1. (A) Gating strategies for the analysis of CD3+CD137+ of two representatives (R/M) HNSCC patients. Lymphocytes were selected according to FSC-A/SSC-A, followed by the selection of live/dead cells (LD) . FSC-H and FSC-A were successively used to identify the single cells. CD3+ cells were then selected by SSC-A/CD3 (BV510). CD3+ cells were finally examined for the expression of CD137 (APC). Fluorescence Minus One (FMO) were used as negative controls for the expression of CD137. The red arrow identifies the population represented in the next graph. (B) The scattered dot plots show the values of $CD8^{+}CD137^{+}$ (left) and $CD4^{+}CD137^{+}$ (right) T cells in responder (R) and non-responder (NR) patients. The horizontal lines corresponded to the median values of each CD137+ population in R and NR patients. Data demonstrated that the $CD8^{+}CD137^{+}$ and $CD4^{+}CD137^{+}$ T cell subsets did not correlate with response to anti-PD1 treatment.
